# COVID-19 in Patients with Melanoma: A Single-Institution Study

**DOI:** 10.3390/cancers16010096

**Published:** 2023-12-24

**Authors:** Amalia Anastasopoulou, Panagiotis T. Diamantopoulos, Panagiotis Kouzis, Maria Saridaki, Konstantinos Sideris, Michael Samarkos, Helen Gogas

**Affiliations:** First Department of Internal Medicine, Laikon General Hospital, National and Kapodistrian University of Athens, 11527 Athens, Greece; pandiamantopoulos@gmail.com (P.T.D.); panagiotiskouzis@yahoo.gr (P.K.); onchocercavolcul@gmail.com (M.S.); kostsideris@gmail.com (K.S.); msamarkos@med.uoa.gr (M.S.); helgogas@gmail.com (H.G.)

**Keywords:** melanoma, COVID-19, COVID-19 severity, immunotherapy, COVID-19 vaccination

## Abstract

**Simple Summary:**

The complexity of possible interactions of baseline patient characteristics and cancer-related factors with the course of COVID-19 is illustrated by the controversial results of studies that assess the severity and outcome of COVID-19 in cancer patients. The present research is a single-institution analysis of 121 melanoma patients with COVID-19. The purpose of our study is to characterize the spectrum of severity and outcome of COVID-19 in melanoma patients. The 30-day mortality rate after COVID-19 infection was low (4.2%). Melanoma stage, treatment receipt and treatment type had no impact on COVID-19 severity and hospitalization risk. Vaccinated patients experienced milder disease compared to the unvaccinated ones. Heart failure and the time period of the infection were independent predictors of severity. The results of the study expand the evidence on the safety of melanoma treatments in light of COVID-19 and will be useful to clinicians treating melanoma patients with COVID-19.

**Abstract:**

We conducted a single-center, non-interventional retrospective study of melanoma patients with COVID-19 (1 March 2020 until 17 March 2023). The cohort was further divided into three groups according to the periods of SARS-CoV-2 variant dominance in Greece. We recorded demographics, comorbidities, vaccination data, cancer diagnosis/stage, types of systemic melanoma treatments, date of COVID-19 diagnosis and survival. We identified 121 patients. The vast majority (87.6%) had advanced disease (stages III or IV). A total of 80.1% of the patients were receiving immune checkpoint inhibitor-based therapies, 92.5% had asymptomatic/mild COVID-19 and 7.4% had moderate/severe/critical disease, while 83.5% contracted COVID-19 during the third period of the pandemic. Sixteen patients (13.2%) were hospitalized for COVID-19 with a median length of stay of 12 days (range: 1–55 days). Advanced age, heart failure, number of comorbidities (≤1 vs. >1), vaccination status and the time period of the infection correlated with more severe COVID-19, whereas only heart failure and time period were independently correlated with severity. The 30-day mortality rate after COVID-19 was 4.2%. With a median follow-up of 340 days post-COVID-19, 17.4% of patients were deceased. In this cohort of melanoma patients with COVID-19, the 30-day mortality rate was low. There was no association between melanoma stage, treatment receipt and type of treatment with COVID-19 severity.

## 1. Introduction

The coronavirus disease 2019 (COVID-19) pandemic has had a major impact on all aspects of life in the past three years. As of April 2023, more than 761 million cases have been reported, while over 6.8 million people have died from complications [1]. The clinical spectrum of the disease ranges widely from an asymptomatic course and mild upper respiratory illness to severe acute respiratory syndrome and death [2]. Advanced age, male sex and co-morbidities, such as obesity, diabetes and cardiovascular disease, have been associated with increased disease severity and mortality [3,4].

There is great interest in the severity and outcome of COVID-19 in patients with cancer. The vast majority of studies that were conducted during the early course of the pandemic, as well as more recent meta-analyses, have shown that cancer patients are at increased risk for severe COVID-19 and death [5,6,7,8], and certain cancer types, particularly hematologic malignancies, head and neck cancer, lung cancer and genitourinary cancers, have been associated with disproportionately worse outcomes [8,9,10]. Cancer patients may be prone to severe COVID-19 due to immunosuppression from cancer itself [11]. Risk might also be influenced by cancer treatment, although results are conflicting for patients who undergo immunotherapy and chemotherapy, as these treatment modalities have been associated with severe disease in some [5,12,13,14,15] but not all studies [10,16,17,18,19]. Moreover, cancer patients are frequently of advanced age and have comorbidities that are associated with higher risk. Further studies of homogeneous patient populations are needed in terms of baseline characteristics and cancer type/stage and treatment in order to evaluate the precise impact of certain cancer types and treatment modalities on the outcome of COVID-19. Moreover, the improvements in the diagnosis and management of COVID-19 that have contributed, among other factors, to a time-dependent decrease in COVID-19 severity and mortality in cancer patients [20], as well as the implementation of vaccination against SARS-CoV-2, underscore the need for studies that cover long periods of the pandemic. 

Melanoma accounts for less than 5% of cutaneous malignancies; however, it is a highly malignant neoplasm that is responsible for >75% of skin cancer-related mortality [21]. Both molecularly targeted treatments (for BRAF mutated cases) and immunotherapy with immune checkpoint inhibitors (ICIs) are first-line treatment options for advanced disease [22]. Evidence on the impact of melanoma and its treatment on COVID-19 severity and survival are scarce and derive mostly from early studies that include small numbers of patients [19,23] and melanoma registry-based studies [24,25,26]. The present study is an analysis of 121 melanoma patients treated at a large academic tertiary referral center in Greece who were diagnosed with COVID-19. The objective of the present study was to characterize the spectrum of severity and outcome of COVID-19 in melanoma patients to identify prognostic factors of severity as well as to point out potential differences across the different stages of the pandemic so far.

## 2. Patients and Methods

### 2.1. Patients

We performed a single-center, non-interventional retrospective study of patients with melanoma who were treated in the Oncology Outpatient Department of the First Department of Internal Medicine at Laikon General Hospital, Athens, Greece, and were diagnosed with SARS-CoV-2 infection. The department in which the study was performed is the largest national melanoma referral center. The study was approved by the Institutional Review Board of the participating center (IRB protocol number: 67/25.01.21). Patient informed consent was waived because of the retrospective nature of the study and the anonymity of data that posed minimal risk to study participants.

We analyzed patients aged ≥18 years with an active melanoma diagnosis who were diagnosed with SARS-CoV-2 infection confirmed by RT-PCR or antigen testing. The study period was set from 1 March 2020 until 17 March 2023. Active disease was defined as detectable disease upon COVID-19 diagnosis or if patients were currently on treatment. The cohort was divided into three groups according to the periods of SARS-CoV-2 variant dominance in Greece: the first period extending from March 2020 until June 2021 (the alpha variant), the second period from July 2021 until December 2021 (the delta variant) and the third period from January 2022 until March 2023 (the omicron variant).

We collected data from medical records for variables concerning demographics, comorbidities, vaccination status, cancer diagnosis and stage, types of systemic melanoma treatments, smoking status, date of COVID-19 diagnosis, and follow-up survival data. Regarding comorbidities, we recorded histories of hypertension, diabetes mellitus, smoking, heart failure (NYHA class ≥ 2), chronic lung disease (COPD, asthma and pulmonary fibrosis), obesity (BMI ≥ 25), chronic kidney disease (with eGFR < 60 mL/min/1.73 m^2^), chronic liver disease (chronic hepatitis, cirrhosis and hepatocellular carcinoma), dementia, and immunosuppressive conditions (other than melanoma and its treatment). Patients with no record of receiving any vaccination against SARS-CoV-2 were classified as unvaccinated, those who had not completed a primary vaccination series (or for whom the time between the last dose of a primary series and COVID-19 onset was ≤14 days) were recorded as partially vaccinated, and patients who had completed a primary series ≥ 14 days before COVID-19 onset were recorded as fully vaccinated. Melanoma stage was classified according to the AJCC, 8th edition [27]. The type of systemic melanoma treatment was defined as ICI-based or non-ICI-based. Patients were classified as untreated if the last dose was received over 6 months before COVID-19 diagnosis.

The primary objective was to evaluate COVID-19 severity and to identify the baseline and melanoma features that were associated with increased severity and hospitalization. COVID-19 severity was categorized as asymptomatic, mild, moderate, severe or critical [28], and was further dichotomized as asymptomatic/mild or moderate/severe/critical. The secondary objectives were to determine oxygen needs and 30-day mortality, as well as mortality throughout the period of follow-up. 

### 2.2. Statistical Analysis

The statistical analysis of the results was performed using the IBM Statistical Package for Social Sciences (SPSS) statistics, version 26 (IBM Corporation, North Castle, NY, USA). Associations between categorical variables were tested with the Pearson chi-squared test (or Fisher’s exact test for categories with fewer than five results), while testing between a categorical variable with two or more levels and not normally distributed continuous variables was performed with the independent-samples Mann–Whitney U test and the Kruskal–Wallis 1-way ANOVA, respectively. Binary logistic regression models were built to assess for associations between categorical variables (risk factors for severe COVID-19) and outcome measures. The models only included variables that proved to be statistically significant in univariate analysis. Median values and a 95% confidence interval (CI) were used in the analyses, while the level of significance for all statistical tests was set at a probability value lower than 5% (2-sided *p* < 0.05).

## 3. Results

### 3.1. Demographic and Clinical Characteristics of Melanoma Patients with COVID-19

We identified 121 patients with melanoma and confirmed COVID-19. Briefly, their median age was 61 years (range: 20–94) and 49.6% were males. Seventy-seven patients (64.1%) had at least one comorbidity. The most common comorbidities were arterial hypertension (49/121, 40.5%) and diabetes mellitus (29/121, 24%), while two patients (1.7%) had a concurrent malignancy and six patients (4.9%) had an immunosuppressive condition (other than malignancy). In terms of vaccination status, 95 patients (78.5%) were vaccinated with at least one dose. More specifically, 26 patients (21.5%) were unvaccinated, 19 (15.7%) were partially vaccinated and 76 patients (62.8%) were fully vaccinated. Regarding melanoma disease status, 70 patients (57.9%) had stage IV melanoma, 31 (25.6%) had stage IIIC/D, 5 (4.1%) had stage IIIA/B, 14 (11.6%) had stage II and 1 (0.8%) had stage I melanoma. Moreover, 106 (87.6%) patients had received treatment for melanoma during the last six months; 60/106 (56.6%) were receiving treatment for metastatic disease, while 46/106 (43.4%) were undergoing adjuvant treatment. The majority of patients (85/106, 80.1%) were receiving ICI-based regimens. Baseline characteristics, vaccination data and melanoma-related characteristics are outlined in Table 1.

### 3.2. COVID-19 Presentation in Melanoma Patients

A total of 112 patients (92.5%) presented with asymptomatic/mild SARS-CoV-2 infection and 9 (7.4%) presented with moderate/severe/critical disease. The vast majority of patients (83.5%) contracted COVID-19 during the third period of the pandemic. Only 15 patients (12.4%) received antiviral prophylaxis after the diagnosis of COVID-19, 9 patients (7.4%) received nirmatrelvir/ritonavir and 6 (5.0%) received a 3-day course of remdesivir. Sixteen patients (13.2%) were hospitalized for COVID-19 infection with a median length of stay (LOS) of 12 days (range: 1–55 days), and 37.5% developed an in-hospital secondary bacterial infection. In terms of oxygen needs, six patients (5%) needed low-flow oxygen supplementation (defined as nasal cannula or a Venturi mask with fixed delivery flow <50%), two (1.7%) needed aggressive non-invasive ventilation (defined as a Venturi mask with fixed flow ≥ 50%, a non-rebreather mask, high-flow nasal oxygen or bi-level positive airway pressure) and one (0.8%) was intubated. Eleven patients (9.1%) had melanoma treatment delays due to COVID-19, and the median duration of delay was 11 days (range: 7–70).

Importantly, 17 patients (14%) contracted a second SARS-CoV-2 infection at a median time of 251 days after the first infection (range: 49–409 days). No patients were hospitalized during the second infection. COVID-19 presentation data for melanoma patients are presented in Table 2.

### 3.3. Correlation of COVID-19 Severity, Hospitalization and Oxygen Needs with Baseline Characteristics

In a univariate analysis, severity correlated with the presence of advanced age (≥65 years), heart failure, number of comorbidities (≤1 versus >1), vaccination status and time period/predominant variant. No correlation of severity with gender, hypertension, diabetes, obesity, smoking, chronic lung disease, chronic kidney/liver disease, other immunosuppression, other malignancy, dementia, primary-series vaccine type and number of doses, or receipt of prophylactic treatment against SARS-CoV-2 was found. Regarding the melanoma-related characteristics, severity was not correlated with melanoma stage, treatment receipt within the last 6 months, treatment setting, number of treatment lines or type of melanoma treatment at COVID-19 infection (ICI-based vs. non-ICI-based). Furthermore, we compared ICI-treated patients with those treated with targeted therapy or untreated patients, and there was also no significant difference in terms of COVID-19 severity (Table 3).

The impact of prophylactic antiviral treatment on COVID-19 severity was not examined, since prophylaxis was not prescribed according to specific criteria. Nevertheless, we performed a second analysis that excluded the patients who received antiviral prophylaxis. We noticed no differences in correlations between patient characteristics and severity in the two analyses, with the exception of vaccination status, which did not correlate with COVID-19 severity in the group of patients who had not received antiviral prophylaxis (Table 3).

The variables found to be correlated with COVID-19 severity were used in a binary logistic regression model that showed that only heart failure and infection period were independently associated with disease severity, as shown in Table 4. Hospitalization risk correlated with the presence of advanced age, heart failure, dementia and >1 comorbidity, as well as the number of vaccine doses and vaccination status and the time period of the infection. No correlation with gender, hypertension, diabetes, obesity, smoking, chronic lung disease, chronic kidney/liver disease, other immunosuppression, other malignancy, type of vaccine primary series, melanoma stage, treatment within six months, treatment setting or type of melanoma therapy (ICI) was found. However, there was a marginally increased risk for hospitalization for patients treated with ICI-based regimens compared to those treated with targeted therapy or those untreated, as depicted in Table 3.

Finally, oxygen need correlated with age, presence of >1 comorbidity, heart failure and vaccination status. The median age of patients in need of oxygen supplementation was 76 (range: 62–94 years), while that of patients not needing oxygen was 59 (20–87), *p* = 0.006. Additionally, 6/40 (15.0%) patients with >1 comorbidity needed oxygen vs. 3/81 (3.7%) with ≤1 comorbidity (*p* = 0.035). Three out of ten (30.0%) patients with heart failure vs. 6/111 (5.4%) patients without heart failure had oxygenation requirements (*p* = 0.027), and 5/26 (19.2%) unvaccinated patients vs. 4/95 (4.2%) partially or fully vaccinated patients needed oxygen (*p* = 0.03). The infection period strongly correlated with oxygenation needs, as 7/13 patients with COVID-19 in the first period vs. 2/13 in the second and 4/101 in the third needed oxygen supplementation (*p* = 0.0001).

### 3.4. Outcome

The 30-day mortality post-COVID-19 diagnosis was 4.2%, and 30-day mortality attributed to COVID-19 was 2.5% (Table 2). Death was not attributed to COVID-19 if the patient died due to melanoma progression or other comorbidities. Eleven patients (9.1%) had a median delay in melanoma treatment due to COVID-19 of 11 days (range: 7–70 days) and two patients (1.7%) discontinued treatment. Increased COVID-19 severity correlated with delay in melanoma treatment; 7/81 patients with asymptomatic or mild disease had delays in treatment compared to 4/9 patients with moderate/severe/critical disease (*p* = 0.002). Furthermore, 5 out of 16 (31.3%) hospitalized patients had delays in melanoma treatment, while only 6/68 (8.8%) non-hospitalized patients had treatment delays (*p* = 0.01). With a median follow-up of 340 days post-COVID-19 (range: 18–861), 17.4% of patients were deceased. Melanoma-related outcomes are illustrated in Table 5.

## 4. Discussion

The present study is the largest single-institution analysis of melanoma patients with COVID-19. No evidence was derived from our study to support that advanced melanoma stage can negatively affect the clinical course of COVID-19. By contrast, advanced stage was found to lead neither to more severe disease nor to an increase in hospitalizations or need for oxygen supplementation. Furthermore, treatment delivery did not affect disease severity, hospitalization or oxygen needs. In our study, the vast majority of on-treatment patients (80.1%) were receiving ICI-based therapies. When comparing ICI-treated patients with those on non-ICI treatment, we found no differences in COVID-19 severity, hospitalization risk or oxygen requirements; however, when we compared ICI-treated patients with patients receiving targeted treatment or those untreated, we found a marginal increase in hospitalization risk, whereas there were no differences among treatment arms in terms of severity. This may reflect physician biases, given that the impact of ICIs on COVID-19 course is an area of controversy, as ICI use may restore the exhausted T-cell responses against SARS-CoV-2 [29,30] but could also exacerbate inflammatory changes leading to severe COVID-19 complications [31]. Nevertheless, our results are in line with those of previous studies in melanoma patients. The GRAVID study by the Spanish Melanoma Group reported that stage and melanoma treatment did not affect prognosis in a cohort of 150 unvaccinated melanoma patients [25]. Similarly, Johnson et al. reported that melanoma therapy, irrespective of modality, was not associated with severity of COVID-19 [26]. Treatment continuation, therefore, should be examined, particularly for asymptomatic and mild cases, considering the potential negative influence of treatment delay in melanoma outcome. In our cohort, only 9.1% of patients had treatment delays, among whom the majority had a more aggressive COVID-19 course and had been hospitalized. 

The median age of patients in our study was 61 years, whereas 47% were younger than 65 years of age. Moreover, only 32.2% had >1 comorbidity. Advanced age (≥65 years), presence of heart failure and >1 comorbidity strongly correlated with increased severity, whereas the necessity for inpatient treatment emerged for patients with a median age of >75 years compared to <60 years for non-hospitalized patients and for patients with heart failure, dementia, >1 comorbidity and hypertension (marginal association). Heart failure was the only coexisting condition that was independently associated with increased COVID-19 severity. Age and certain pre-existing comorbidities, as well as multimorbidity, have been associated with COVID-19 severity and mortality [32]. However, studies have produced inconsistent results, and the establishment of causality as well as the identification of underlying mechanisms is methodologically difficult [32]. Until further data become available, particular attention should be given to melanoma patients with heart failure who present with COVID-19.

When comparing the three different periods of COVID-19, we observed that the vast majority of patients (83.5%) contracted COVID-19 during the third period of the pandemic, when omicron and its subvariants were the most dominant versions of SARS-CoV-2 in Greece. This may be attributed to the increased transmissibility of the omicron variant and its sublineages and increased immune evasion [33,34] as well as to changes in national public-health strategies over time. Infection during the third period was independently associated with less severe COVID-19. Moreover, hospitalization risk and oxygenation needs decreased during the third period compared to the early phases of the pandemic. Meng et al. showed that omicron has a suboptimal fusion capacity in comparison to other variants [35], and numerous clinical studies have reported that omicron results in less severe disease and fewer hospitalizations [36,37,38,39,40,41,42], even after adjustment for potential confounders, such as vaccination status [38,39,40], age and frailty [40], suggesting that omicron and its subvariants may exhibit a reduced inherent virulence. This may contribute to the decreased COVID-19 severity and hospitalization rate observed in our study compared to studies that were carried out during earlier stages of the pandemic [26,43].

In our cohort, 78.5% of patients were vaccinated, either partially or fully, against SARS-CoV-2 prior to COVID-19 infection. Vaccination with at least one dose resulted in an inpatient rate of 9.5%, whereas more than 25% of unvaccinated patients were hospitalized. Additionally, a five-fold increase in rates of oxygenation need was observed among unvaccinated patients compared to vaccinated ones. Surprisingly, vaccination status did not independently correlate with severity. Our results highlight the need for additional studies in order to determine the exact impact of vaccination status (as well as the impact of bivalent vaccines, which was not studied in the present analysis) on COVID-19 severity in melanoma patients in the omicron era. 

This study has several limitations and strengths. First, this was a retrospective, single-institution study and is probably prone to recall and selection bias toward advanced stage and an active treatment population. Another limitation of our study is that the majority of patients were treated with ICIs, and no safe conclusions can be drawn regarding the exact impact of other treatment modalities (such as targeted therapies and chemotherapy) on COVID-19 severity. Patient-reporting of comorbidities and in-hospital treatment modalities of COVID-19 in some cases could also have limited our results. Moreover, we did not analyze the potential effect of prophylactic antiviral treatment on COVID-19 severity. On the other hand, the strength of the present study is that it is the largest single-institution analysis with a well-characterized melanoma population. Moreover, the study period covered all the stages of the pandemic so far. Nevertheless, further studies assessing more heterogeneous melanoma patient populations are needed in order to clarify the exact impact of patient-, treatment- and melanoma-related factors on COVID-19 severity.

## 5. Conclusions

In summary, melanoma stage, treatment receipt and type of treatment had no impact on COVID-19 severity in our study. Heart failure was the only comorbid condition independently associated with more severe disease. Although vaccinated patients experienced milder disease compared to the unvaccinated ones, vaccination status was not an independent predictor of COVID-19 severity. Patients who presented with COVID-19 during the third period of the pandemic had less severe disease compared to patients who contracted COVID-19 during the early phases of the pandemic, independent of vaccination and patient characteristics. Our study expands the evidence on the safety of melanoma treatment modalities in light of COVID-19.

## Figures and Tables

**Table 1:** Baseline patient characteristics and vaccination status.

GENERAL CHARACTERISTICS	
Number of patients, N (%)	121 (100)
Gender (male), N (%)	60 (49.6)
Age (years), median (range)	61.0 (20.0–94.0)
≥65 years	52 (43.0)
<65 years	69 (57.0)
FACTORS PREDISPOSING TO SEVERE COVID-19	
Hypertension, N (%)	49 (40.5)
Diabetes mellitus, N (%)	29 (24.0)
Obesity (≥30 kg/m^2^), N (%)	11 (9.1)
Smoking, N (%) (data available for 51 patients)	
Yes	10 (8.3)
No	42 (34.7)
Heart failure (NYHA class ≥ 2), N (%)	10 (8.3)
Chronic lung disease, N (%)	10 (8.3)
Chronic kidney disease, N (%)	3 (2.5)
Chronic liver disease, N (%)	3 (2.5)
Dementia, N (%)	4 (3.3)
Other malignancy, N (%)	2 (1.7)
Other immunosuppression, N (%)	6 (4.9)
Type of other immunosuppression, N (%)	
Immune-related hepatitis	4 (3.3)
Immune-related nephritis	1 (0.8)
Sickle cell disease	1 (0.8)
Number of comorbidities, N (%)	
0	43 (35.5)
1	38 (31.4)
2	24 (19.8)
3	13 (10.7)
4	2 (1.6)
VACCINATION DATA	
Vaccinated with at least one dose, N (%)	95 (78.5)
Vaccination status, N (%)	
Unvaccinated	26 (21.5)
Partially vaccinated	19 (15.7)
Completed primary vaccination	76 (62.8)
Primary series vaccine type, N (%)	
BioNTech/Pfizer	76 (62.8)
Moderna COVID-19	9 (7.4)
Oxford/AstraZeneca ChAdOx1-S	9 (7.4)
J&J/Janssen COVID-19	1 (0.8)
Primary series vaccine type, N (%)	
mRNA vaccine	85 (70.2)
Non-mRNA vaccine	10 (8.2)
MELANOMA-RELATED CHARACTERISTICS	
Melanoma stage at first COVID-19 diagnosis, N (%)	
I	1 (0.8)
II	14 (11.6)
IIIA/B	5 (4.1)
IIIC/D	31 (25.6)
IV	70 (57.9)
Previous lines of treatment at first COVID-19 diagnosis, N (%)	
0	7 (5.8)
1	77 (63.6)
2	19 (15.7)
3	13 (10.7)
4	2 (1.7)
5	1 (0.8)
>5	1 (0.8)
Melanoma treatment, N (%)	
Within the last 6 months	106 (87.6)
Untreated	14 (11.6)
Treatment setting, N (%) (among 106 patients)	
Adjuvant	46 (43.4)
Metastatic	60 (56.6)
Treatment type upon COVID-19 diagnosis, N (%) (among 106 patients)	
ICI-based regimens	85 (80.1)
Non-ICI-based regimens	18 (17.0)
Melanoma status upon COVID-19, N (%)	
Unknown	1 (0.8)
SD	41 (33.9)
PR	4 (3.3)
CR	6 (5.0)
PD	19 (15.7)
NED	45 (37.2)

Abbreviations: N, number; SD, stable disease; PR, partial response; CR, complete response; PD, progressive disease; NED, no evidence of disease.

**Table 2:** COVID-19 presentation.

COVID-19—FIRST INFECTION	
Time from latest vaccine dose until COVID-19 diagnosis (days), median (range)—for vaccinated patients	159 (6–527)
SARS-CoV-2 time period at COVID-19 diagnosis, N (%)	
First period	7 (5.8)
Second period	13 (10.7)
Third period	101 (83.5)
Hospitalization, N (%)	16 (13.2)
In-hospital infection, N (%) (only among hospitalized patients)	6 (37.5)
Duration of hospitalization (days), median (range)	12 (1–55)
Prophylactic COVID-19 treatment, N (%)	
None	106 (87.6)
Remdesivir (3-day regimen)	6 (5.0)
Nirmatrelvir/ritonavir	9 (7.4)
COVID-19 treatment, N (%)	
None	108 (89.3)
Dexamethasone/remdesivir	13 (10.7)
Five-variable COVID-19 severity, N (%)	
Asymptomatic	30 (24.8)
Mild	82 (67.8)
Moderate	6 (5.0)
Severe	2 (1.7)
Critical	1 (0.8)
COVID-19 severity (dichotomized), N (%)	
Asymptomatic/mild	112 (92.5)
Moderate/severe/critical	9 (7.4)
Outcome at 30 days after COVID-19 diagnosis, N (%)	
Alive	116 (95.9)
Death due to other conditions	2 (1.7)
Death due to COVID-19	3 (2.5)
COVID-19—SECOND INFECTION	
Second COVID-19 infection, N (%)	17 (14.0)
Time from first to second infection (days), median (range)	251 (49–409)
SARS-CoV-2 time period at second infection	
First period	0 (0.0)
Second period	1 (0.8)
Third period	16 (13.2)
Hospitalization during second COVID-19 infection, N (%)	0 (0.0)

Abbreviations: N, number.

**Table 3:** Correlations between baseline characteristics and COVID-19 severity and hospitalization: univariate analysis.

	COVID-19 Two-Variable Severity			COVID-19 Severity in Patients without Prophylactic Treatment (N = 106)			Hospitalization		
	Asymptomatic/Mild	Moderate/ Severe/ Critical	*p*	Asymptomatic/Mild	Moderate/ Severe/ Critical	*p*	No	Yes	*p*
Gender			0.311			0.365			0.616
Male	57	3		51	4		53	7	
Female	55	6		50	1		52	9	
Age			0.028			0.009			0.005
<65	67	2		63	0		65	4	
≥65	45	7		38	5		40	12	
Hypertension			0.096			0.355			0.054
No	69	3		65	2		66	6	
Yes	43	6		36	3		39	10	
Diabetes			0.494			0.594			0.173
No	86	6		77	3		82	10	
Yes	26	3		24	2		23	6	
Obesity			0.862			1.000			0.585
No	97	8		86	5		92	13	
Yes	10	1		10	0		9	2	
Heart Failure			0.005			0.034			0.009
No	105	6		96	3		99	12	
Yes	7	3		5	2		6	4	
Chronic lung disease			0.747			0.397			0.753
No	103	8		92	4		96	15	
Yes	9	1		9	1		9	1	
Smoking			0.721			0.506			0.685
No	36	6		33	2		31	11	
Yes	9	1		8	1		8	2	
Chronic kidney disease			0.564			1.000			0.427
No	108	9		99	5		101	16	
Yes	4	0		2	0		4	0	
Chronic liver disease			0.619			1.000			0.494
No	109	9		98	5		102	16	
Yes	3	0		3	0		3	0	
Immunosuppression			0.476			0.583			0.327
No	106	9		82	5		99	16	
Yes	6	0		19	0		6	0	
Other malignancy			0.686			1.000			0.578
No	110	9		99	5		103	16	
Yes	2	0		2	0		2	0	
Dementia			0.173			0.178			0.027
No	109	8		98	4		103	14	
Yes	3	1		3	1		2	2	
Number of comorbidities			0.026			0.036			0.034
≤1	78	3		71	1		74	7	
>1	34	6		30	4		31	9	
Primary-series vaccine type			1.000			1.000			0.279
mRNA	84	4		70	3		76	9	
Non-mRNA	10	0		10	0		10	0	
Number of vaccine doses			0.137			0.757			0.047
0	21	5		21	2		19	7	
1	2	0		1	0		1	1	
2	19	1		17	0		17	3	
3	62	3		57	3		60	5	
4	8	0		5	0		8	0	
Vaccination status			0.035			0.222			0.017
Unvaccinated	21	5		15	4		19	7	
Partially	18	1		15	2		15	4	
Fully	73	3		68	2		71	4	
Vaccination status			0.010			0.296			0.02
Unvaccinated	21	5		21	2		19	7	
Partially/fully	91	4		80	3		86	9	
Melanoma stage			0.563			1.000			0.328
I, II, III	48	3		47	2		46	5	
IV	63	6		53	3		58	11	
Τreatment			0.257			1.000			0.118
Within 6 months	97	9		87	5		90	16	
>6 months	14	0		13	0		14	0	
Treatment setting			0.180			1.000			0.107
Metastatic	53	7		45	3		48	12	
Adjuvant	44	2		42	2		42	4	
Number of treatment lines			0.372			0.628			0.166
0	6	0		6	0		6	0	
1	74	4		67	3		69	9	
2	15	4		13	1		15	4	
3	12	1		11	1		11	2	
4	2	0		2	0		2	0	
5	1	0		1	0		1	0	
Type of treatment			0.354			0.580			0.068
ICI-based	76	9		68	5		69	16	
Non-ICI-based	18	0		16	0		18	0	
Type of treatment (ICI-based vs. targeted vs. none)			0.189			1.000			0.042
ICI-based	76	9		68	5		69	16	
Targeted	15	0		13	0		16	0	
None	14	0		13	0		14	0	
Period of infection			<0.001			0.277			0.001
First	4	3		4	1		3	4	
Second	11	2		10	0		10	3	
Third	97	4		87	4		92	9	

The chi-squared test (or Fisher’s exact test when appropriate) was used for all comparisons.

**Table 4:** Binary logistic regression model comprising all variables that proved to be correlated with COVID-19 severity.

			95% Confidence Interval for Hazard Ratio	
Covariate	*p*	Hazard Ratio	Lower Bound	Upper Bound
Age ≤ 65 years	0.179	3.992	0.530	30.071
Unvaccinated	0.161	0.210	0.024	1.861
Comorbidities ≤ 1	0.360	2.776	0.312	24.685
Absence of heart failure	0.038	10.973	1.136	106.022
SARS-CoV-2 period (first)	0.03	0.057	0.004	0.758
SARS-CoV-2 period (second)	0.035	0.090	0.010	0.848
SARS-CoV-2 period (third)	-	-	-	-

Binary logistic regression models including all variables proven to be statistically correlated with COVID-19 severity were built.

**Table 5:** Melanoma-related outcomes.

Melanoma status at COVID-19, N (%)	
Unknown	1 (0.8)
SD	41 (33.9)
PR	4 (3.3)
CR	6 (5.0)
PD	19 (15.7)
NED	45 (37.2)
Treatment delay due to COVID-19, N (%)	11 (9.1)
Delay duration (days), median (range)	11 (7–70)
Treatment discontinuation due to COVID-19, N (%)	2 (1.7)
Disease status at 3 months post-COVID-19, N (%)	
Unknown	1 (0.8)
SD	27 (22.3)
PR	4 (3.3)
CR	6 (5.0)
PD	41 (33.9)
NED	39 (32.2)
Survival status at last follow-up, N (%)	
Alive	100 (82.6)
Deceased	21 (17.4)
Survival from COVID-19 diagnosis (days), median (95% CI)	340 (18–861)

Abbreviations: SD, stable disease; PR, partial response; CR, complete response; PD, progressive disease; NED, no evidence of disease.

## Data Availability

Patient data are available upon reasonable request. For original data, please contact amanastasop@yahoo.gr.
